# Clinical Nomograms to Predict Stone-Free Rates after Shock-Wave Lithotripsy: Development and Internal-Validation

**DOI:** 10.1371/journal.pone.0149333

**Published:** 2016-02-18

**Authors:** Jung Kwon Kim, Seung Beom Ha, Chan Hoo Jeon, Jong Jin Oh, Sung Yong Cho, Seung-June Oh, Hyeon Hoe Kim, Chang Wook Jeong

**Affiliations:** 1 Department of Urology, Seoul National University Hospital, Seoul, Korea; 2 Department of Urology, Seoul National University Bundang Hospital, Seongnam, Korea; 3 Department of Urology, Seoul National University Boramae Medical Center, Seoul, Korea; Sun Yat-sen University, CHINA

## Abstract

**Purpose:**

Shock-wave lithotripsy (SWL) is accepted as the first line treatment modality for uncomplicated upper urinary tract stones; however, validated prediction models with regards to stone-free rates (SFRs) are still needed. We aimed to develop nomograms predicting SFRs after the first and within the third session of SWL. Computed tomography (CT) information was also modeled for constructing nomograms.

**Materials and Methods:**

From March 2006 to December 2013, 3028 patients were treated with SWL for ureter and renal stones at our three tertiary institutions. Four cohorts were constructed: Total-development, Total-validation, CT-development, and CT-validation cohorts. The nomograms were developed using multivariate logistic regression models with selected significant variables in a univariate logistic regression model. A C-index was used to assess the discrimination accuracy of nomograms and calibration plots were used to analyze the consistency of prediction.

**Results:**

The SFR, after the first and within the third session, was 48.3% and 68.8%, respectively. Significant variables were sex, stone location, stone number, and maximal stone diameter in the Total-development cohort, and mean Hounsfield unit (HU) and grade of hydronephrosis (HN) were additional parameters in the CT-development cohort. The C-indices were 0.712 and 0.723 for after the first and within the third session of SWL in the Total-development cohort, and 0.755 and 0.756, in the CT-development cohort, respectively. The calibration plots showed good correspondences.

**Conclusions:**

We constructed and validated nomograms to predict SFR after SWL. To the best of our knowledge, these are the first graphical nomograms to be modeled with CT information. These may be useful for patient counseling and treatment decision-making.

## Introduction

The worldwide incidence and prevalence of urinary stones is increasing [1], and accordingly, several guidelines for the treatment of patients with urinary stones have been formulated. Despite the advances of endourologic stone removal techniques, such as ureteroscopy (URS) and percutaneous nephrolithostomy (PNL), shock-wave lithotripsy (SWL) remains one of the first line treatments for most uncomplicated upper urinary tract calculi [2, 3].

A large number of studies investigating the efficacies and outcomes of these treatments, however, report varying results and wide ranges for stone-free rates (SFRs). The 2007 guidelines for the management of ureteral calculi, constructed by the EAU/AUA panel, showed that the ranges of SFRs after URS and SWL differed in more than 70% according to location of stone, maximal stone diameter, and patient age [3]. In this regard, validated prediction models for SFR are still needed for patient counseling and decision-making in treatment strategies.

Previous studies have developed various nomograms for each treatment method including URS and PNL [4, 5]. In recent studies, nomograms to predict SFR after SWL have also been developed [6, 7]. These nomograms, however, are too complex to use as it is non-graphical [6], and is not continuous variable based nomogram [7]. In addition, these nomograms neglect much of the information that can be derived from computed tomography (CT) imaging.

In the present study, we aimed to develop convenient nomograms predicting SFRs after the first and within the third session of SWL as the initial and sole treatment. CT information was also modeled for constructing the nomograms.

## Materials and Methods

### Ethics statement

The Institutional Review Boards (IRBs) of the Seoul National University Hospital (SNUH), Seoul National University Bundang Hospital (SNUBH), and Seoul National University Boramae Medical Center (SNUBMC) approved this study (Approval number: SNUH, H-1503-070-656; SNUBH, B-1503-292-116; SNUBMC, 26-2015-34). As the present study was carried out retrospectively, written informed consent from patients was waived by the IRBs. Personal identifiers were completely removed and the data were analyzed anonymously. Our study was conducted according to the ethical standards laid down in the 1964 Declaration of Helsinki and its later amendments.

### Study cohort

From March 2006 to December 2013, a cohort of 3,274 patients, who were treated with SWL as the initial, sole treatment for ureter and renal stones at our three tertiary institutions (SNUH, SNUBH, and SNUBMC), was included in the analysis. Clinical data in the medical records were retrospectively reviewed. The urinary stones were diagnosed by using Kidneys, Ureters and Bladder (KUB) and/or CT. Patients with a stone size >25mm, or who had bladder stone(s), or were managed with combination or adjuvant therapy, or lost to follow up, were excluded from this study; a total of 3,028 cases were analyzed.

**Fig 1:**
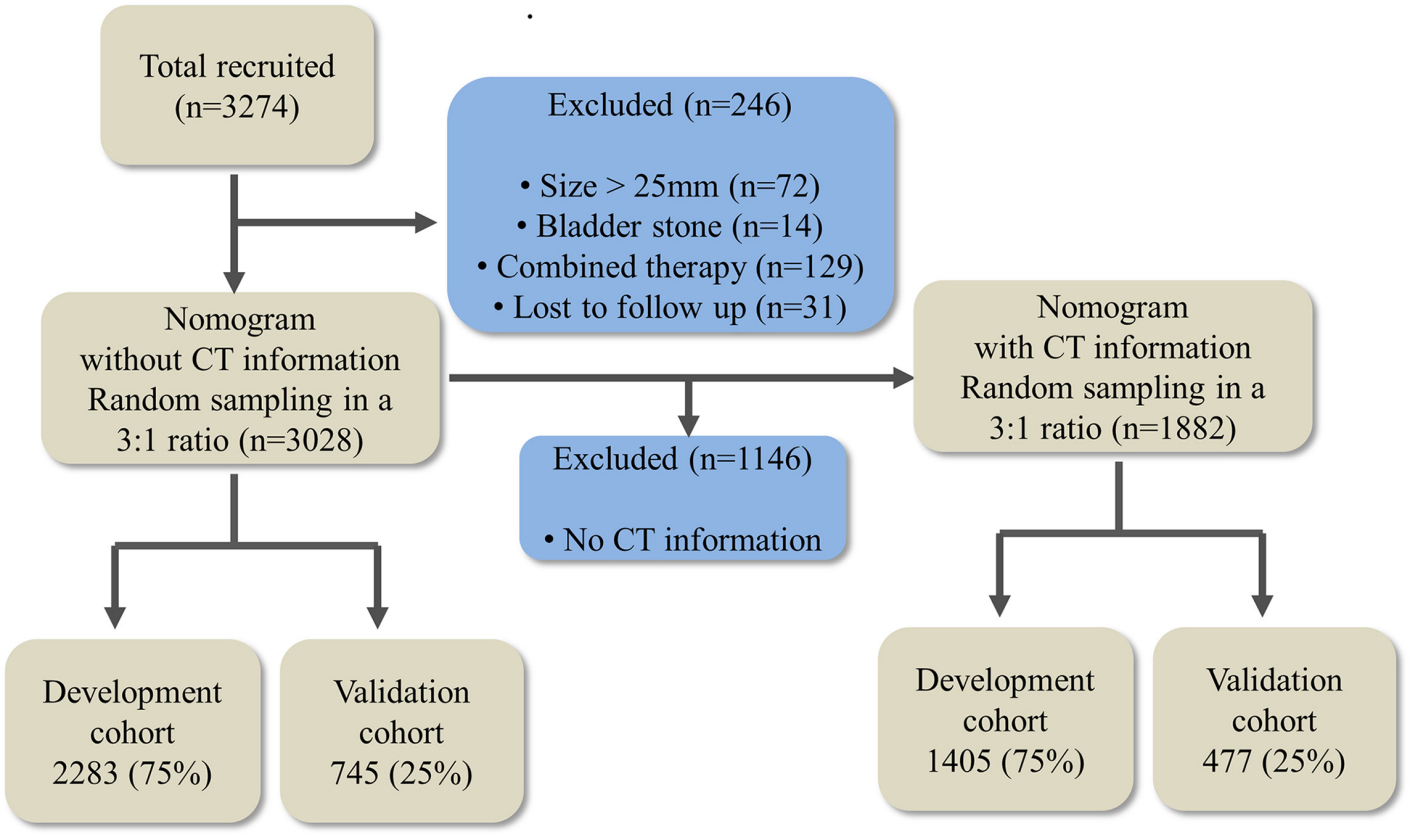
Study flowchart for patient selection.

Four cohorts were constructed: the Total-development and validation cohorts, and the CT-development and validation cohorts. The Total-development cohort, to obtain nomograms predicting the SFRs after first and within third session of SWL, was constructed by random sampling of 75% (2,283) of the 3,028 patients, and the Total-validation cohort was constructed with the remaining 25% (745) of patients. For models with CT information, the CT-development and CT-validation cohorts were constructed with 1,882 patients who underwent CT for stone evaluation, in the same manner (Fig 1).

### SWL methods

SWL was performed with the Sonolith Praktis electroconductive lithotripter (EDAP TMS S.A.,Lyons, France) at SNUH, and with the Dornier Delta Compact (Dornier MedTech, Wessling, Germany) at SNUBH and SNUBMC. The unified shock wave rate and shock wave energy protocol were utilized across our three institutions. The shock-wave rate was gradually increased in every 500 shocks from 60SWs/min up to 120SWs/min, and the mean value was 110SWs/min. Shock-wave energy was started at 5kV and increased to a tolerable level [8], with mean value of 20kV. The mean number of shocks was approximately 3,000 and maximum number of shocks was limited to 5,000. Stone-free, as the primary outcome of this study, was defined as no fragments detected on KUB and/or CT within 3 months after SWL.

### Statistical analysis

The following variables were included in the analysis to predict the treatment outcome after SWL: sex, age, body mass index (BMI), laterality of stone, location of stone, number of stones, stone length (maximal and minimal diameter), skin-to-stone distance (SSD), mean Hounsfield unit (HU) and grade of hydronephrosis (HN). The maximal and minimal diameters of the stone(s) were measured by using KUB and/or CT, while SSD, mean HU, and grade of HN were identified by using CT. The stone diameters, SSD, and HU were determined from usual techniques; mean HU was measured by using an ellipse constructed to calculate the mean HU value and fit completely within the stone with the Ellipse ROS tool in the bone windows on cross-sectional CT scan, and SSD was measured at 0, 45, and 90 degrees using the electronic caliper [9–11]. Subgroup analysis according to the stone location (renal vs. ureter) was conducted to investigate whether the SFRs differed between two groups. A univariate logistic regression model was used to determine statistically significant variables that were then included in multivariate logistic regression model. The nomograms were developed using this final multivariate logistic regression model with selected significant variables. The summary statistic used to evaluate the predictive discrimination of the nomograms was the area under the curve (AUC) [12]. As is well known, the value of the AUC is the same as that yielded by the concordance index (c-index) in a logistic regression model. The maximum value of the AUC is 1.0, indicating a perfect discrimination, whereas 0.5 indicates a random chance to correctly discriminate the outcome with the model [13]. The calibration plots were generated using 200 bootstrapping samples to analyze the consistency of prediction between the predicted probability and the actual outcome. The results are expressed as the mean (95% confidence interval) or n (%). Statistical significance was considered as P < 0.05. All statistical analyses were performed using R for Windows, ver. 3.0.1 with the ‘rms’ package. (R Foundation for Statistical Computing, Vienna, Austria).

## Results

### Patients and stone outcomes

Baseline patient demographics and stone characteristics for the total study population and each development cohort are shown in Table 1. The mean age was 53.3 ± 13.9 years, and 1,906 (62.9%) patients were men. Mean body mass index (BMI) was 24.8 ± 3.6 kg/m^2^. The mean maximal and minimal stone diameter was 8.3 ± 4.0 mm and 5.5 ± 2.6 mm, and 1,314 (43.4%) stones were renal stones. The mean HU was 570.6 ± 288.2 in the CT-development cohort. The SFR after the first and within the third session was 48.3% and 68.8%, respectively. There were no significant differences in any of the other measured parameters between the total study population and each development cohorts. Comparisons of each Total- and CT- development and validation cohorts also had no significant differences (data not shown).

**Table 1:** Baseline characteristics of the total cohort and each development cohort.

	Number or Mean ± SD		
	Total cohort N = 3028	Total-development cohort N = 2283	CT-development cohort N = 1405
Sex (%)			
men	1906 (62.9)	1442 (63.2)	864 (61.5)
women	1122 (37.1)	841 (36.8)	541 (38.5)
Age (year)	53.3 ± 13.9	53.3 ± 14.0	54.4 ± 14.3
BMI (kg/m^2^)	24.8 ± 3.6	24.8 ± 3.6	24.7 ± 3.6
Laterality, Right (%)	1429 (47.2)	1070 (46.9)	649 (46.2)
Location of stone (%)			
upper calyx	231 (7.5)	165 (7.2)	106 (7.5)
mid calyx	348 (11.3)	240 (10.5)	164 (11.7)
lower calyx	580 (18.9)	440 (19.3)	274 (19.5)
pelvis	155 (5.0)	111 (4.9)	84 (6.0)
UPJ	184 (6.0)	144 (6.3)	92 (6.5)
upper ureter	945 (30.8)	718 (31.4)	389 (27.7)
mid ureter	375 (12.2)	274 (12.0)	191 (13.6)
lower ureter	253 (8.2)	191 (8.4)	105 (7.5)
Stone number	1.3 ± 0.7	1.3 ± 0.7	1.3 ± 0.7
Maximal diameter (mm)	8.3 ± 4.0	8.3 ± 4.0	8.6 ± 4.1
Minimal diameter (mm)	5.5 ± 2.6	5.5 ± 2.6	5.6 ± 2.6
CT image (%)	1882 (61.1)		
Hounsfield Unit			570.6 ± 288.2
Skin-to-stone distance (mm)			
horizontal (0 degree)			103.8 ± 52.4
oblique (45 degree)			101.9 ± 62.8
vertical (90 degree)			94.3 ± 44.2
Hydronephrosis (%)			
no			663 (47.1)
Grade 1			351 (25.0)
Grade 2			226 (16.0)
Grade 3			101 (7.2)
Grade 4			64 (4.6)
Stone free after 1^st^ session of SWL (%)	1462 (48.3)	1113 (48.8)	697 (49.6)
Stone free within 3^rd^ session of SWL (%)	2082 (68.8)	1582 (69.3)	960 (68.3)

BMI; body mass index, SWL; shock-wave lithotripsy, UPJ; ureteropelvic junction

### Nomogram development

Univariate analysis showed that sex, location of stone, number of stones, and maximal stone diameter were significant predictors of SFR in the Total-development cohort, for both after the first and within the third session of SWL. Analysis of CT-development cohort showed that HU and grade of HN were additional significant predictors. When these variables were entered into the multivariate logistic regression model, all of the factors except sex were significantly associated with SFR (Table 2).

**Table 2:** Multivariate logistic regression models in the development cohorts. Panel A represents stone-free after the first session of Shock-wave lithotripsy (SWL) and panel B shows stone-free within the third session of SWL.

A						
	OR	95% CI	*p-value*	OR	95% CI	*p-value*
	stone-free after first session of Shock-wave lithotripsy					
	Total-development cohort			CT-development cohort		
Sex (M/F)	1.143	0.948–1.378	0.163	1.117	0.868–1.436	0.390
Location						
Upper & mid calyx	1.000	(reference)	0.000	1.000	(reference)	0.000
lower calyx	0.736	0.546–0.994	0.046	0.595	0.402–0.881	0.010
pelvis	0.556	0.334–0.957	0.034	0.673	0.356–1.273	0.223
UPJ	1.702	1.129–2.566	0.011	1.835	1.039–3.242	0.036
upper & mid ureter	1.955	1.515–2.523	0.000	2.064	1.385–3.076	0.000
lower ureter	1.093	0.758–1.576	0.633	0.858	0.494–1.491	0.586
Stone number						
1	1.000	(reference)	0.017	1.000	(reference)	0.000
2–3	0.713	0.546–0.930	0.013	0.508	0.363–0.710	0.000
≥ 4	0.608	0.327–1.131	0.116	0.666	0.283–1.564	0.350
Maximal diameter	0.851	0.828–0.874	0.000	0.925	0.892–0.958	0.000
Hounsfield unit				0.998	0.997–0.998	0.000
Hydronephrosis						
No				1.000	(reference)	0.174
Gr1				1.065	0.740–1.532	0.734
Gr2				0.843	0.565–1.258	0.402
Gr3				0.818	0.509–1.315	0.407
Gr4				0.469	0.231–0.953	0.036
B						
	OR	95% CI	*p-value*	OR	95% CI	*p-value*
	stone-free after first session of Shock-wave lithotripsy					
	Total-development cohort			CT-development cohort		
Sex (M/F)	1.113	0.902–1.374	0.316	1.180	0.891–1.564	0.248
Location						
Upper & mid calyx	1.000	(reference)	0.000	1.000	(reference)	0.000
lower calyx	0.779	0.578–1.051	0.102	0.528	0.356–0.785	0.002
pelvis	1.005	0.634–1.594	0.984	0.913	0.502–1.660	0.766
UPJ	1.998	1.279–3.121	0.002	2.394	1.224–4.683	0.011
upper & mid ureter	2.870	2.164–3.805	0.000	2.807	1.785–4.415	0.000
lower ureter	1.714	1.127–2.609	0.012	1.272	0.685–2.360	0.446
Stone number						
1	1.000	(reference)	0.000	1.000	(reference)	0.002
2–3	0.753	0.570–0.994	0.046	0.549	0.387–0.778	0.001
≥ 4	0.263	0.143–0.482	0.000	0.494	0.205–1.189	0.116
Maximal diameter	0.888	0.866–0.910	0.000	0.949	0.916–0.984	0.005
Hounsfield unit				0.998	0.998–0.999	0.000
Hydronephrosis						
No				1.000	(reference)	0.045
Gr1				0.768	0.808–1.848	0.343
Gr2				0.724	0.490–1.205	0.251
Gr3				0.467	0.424–1.237	0.237
Gr4				0.998	0.230–0.950	0.035

CI; confidence interval, OR; odds ratio, UPJ; ureteropelvic junction

**Fig 2:**
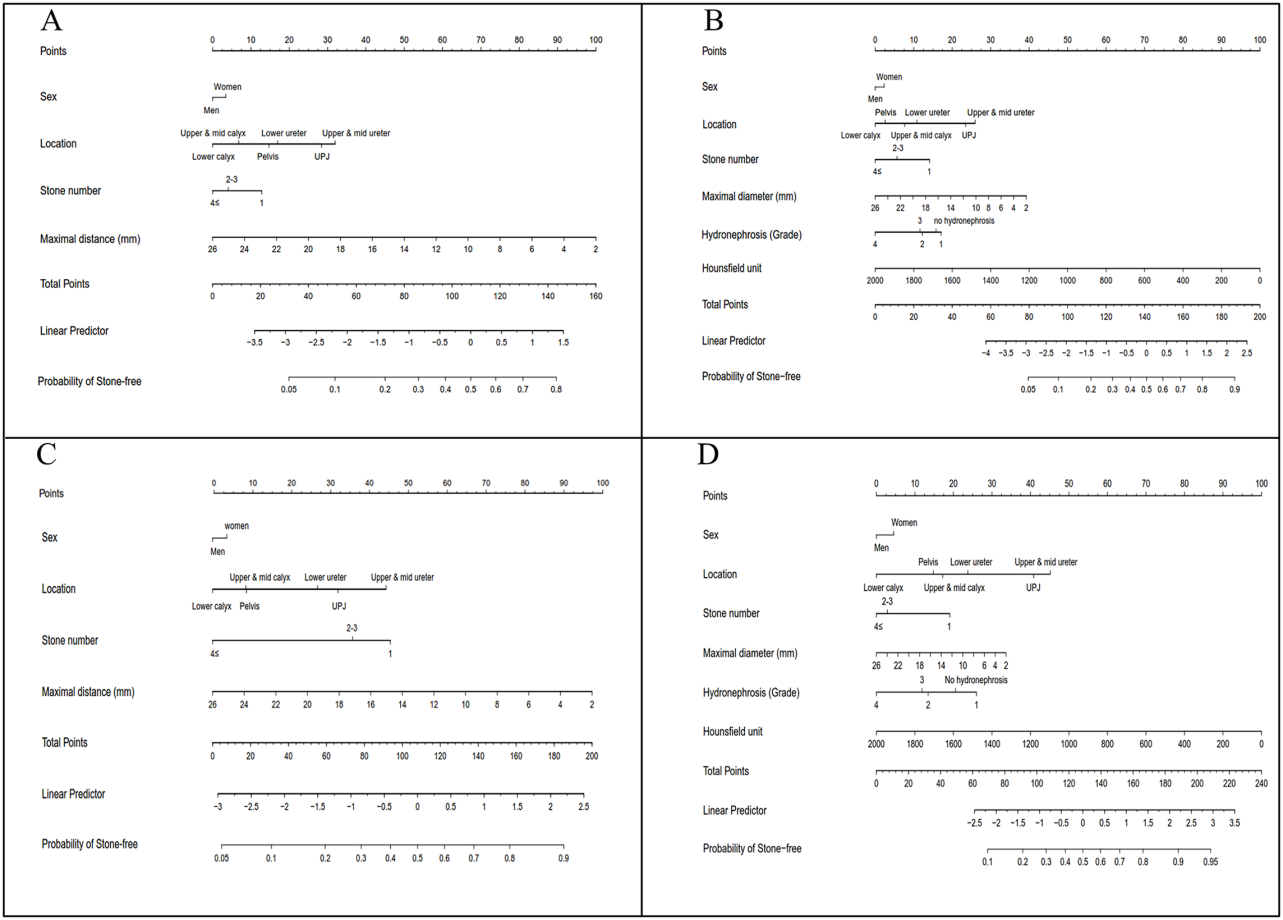
Nomograms predicting stone-free rates. (A) after the first session of shock-wave lithotripsy (SWL) without CT information, (B) after the first session of SWL with CT information, (C) within the third session of SWL without CT information, (D) within the third session of SWL with CT information.

The graphical nomograms predicting SFR after the first and within the third session of SWL, both with and without CT information, were constructed based on the final multivariate logistic regression model (Fig 2).

### Nomogram validation

Regarding performance of the nomograms, for the Total-development cohort, high c-indices were reported for predicting both SFR after the first and within the third session of SWL (0.712; 95% confidence interval [CI]: 0.691–0.734 and 0.723; 95% CI: 0.700–0.747, respectively). Additionally, higher c-indices compared to those of the Total-development cohort were reported in nomograms for the CT-development cohort (0.755; 95% CI: 0.729–0.781 and 0.756; 95% CI: 0.727–0.785, respectively). In the validation cohorts, the predictive accuracy of the Total-validation cohort nomograms, for both SFR after the first and within the third session of SWL, were similar to that of the Total-development cohort with c-indices of 0.710 (95% CI: 0.673–0.748) and 0.714 (95% CI: 0.673–0.755), respectively. In the CT-validation cohort, both sets were also similar to the CT-development cohort with c-indices of 0.744 (95% CI: 0.699–0.789) and 0.740 (95% CI: 0.692–0.788), respectively (Table 3).

**Table 3:** The concordance index (c-index) yielded by AUC to evaluate the predictive discrimination of the nomograms.

	After first session of SWL (95% CI)	Within third session of SWL (95% CI)
Total-development cohort	0.712 (0.691–0.734)	0.723 (0.700–0.747)
Total-validation cohort	0.710 (0.673–0.748)	0.714 (0.673–0.755)
CT-development cohort	0.755 (0.729–0.781)	0.756 (0.727–0.785)
CT-validation cohort	0.744 (0.699–0.789)	0.740 (0.692–0.788)

AUC; area under the curve, CI; confidence interval

**Fig 3:**
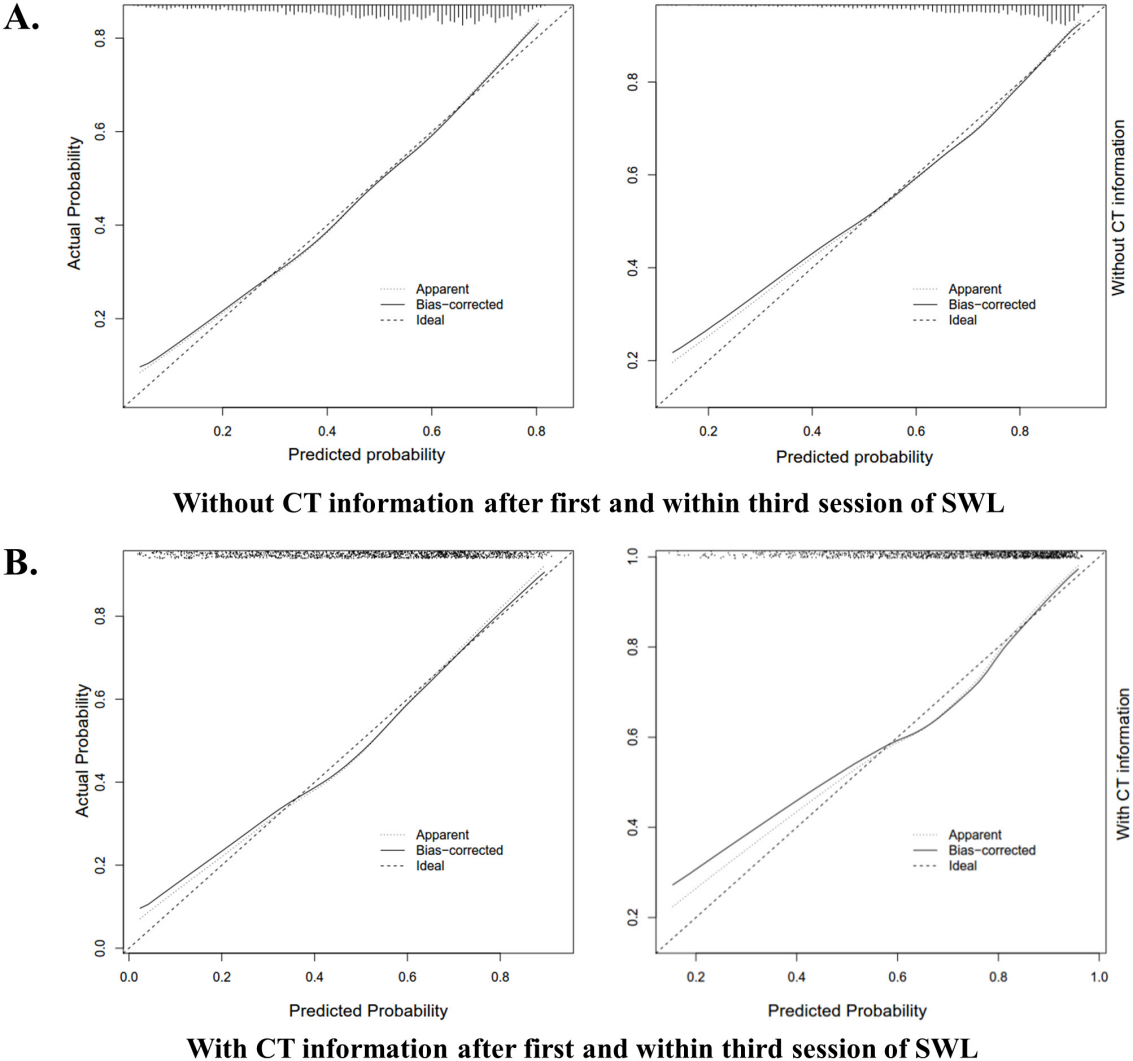
Calibration plots for nomograms predicting stone-free rates after the first and within the third session of SWL with and without CT information. (A) Total-development cohort, (B) CT-development cohort.

The calibration plot showed that all nomograms had good correspondence between the predicted and actual probability of SFR after the first and within the third session of SWL, with and without CT information, indicating that these were well calibrated (Fig 3, S1 Fig).

## Discussion

Various nomograms are utilized for patient counseling and decision making in treatment strategies within diverse areas of urology [14–16]. For urolithiasis, some nomograms are utilized in clinical practice [4, 5], and even nomograms predicting the SFR after SWL have been developed [6, 7]. These existing nomograms, however, carry limitations such as neglect of much of the information derived from CT imaging, and difficulty in usage as it is non-graphical [6], and is not continuous variable based nomogram [7].

In this study, we further investigated upon these characteristics and developed nomograms, taking into account CT information, and predicting the success rate after a single session of SWL and within the third session of SWL. Our nomograms, when used in clinical practice, can be anticipated to take into account various clinical parameters including CT information. Also, using the AUC to evaluate the accuracy of our nomograms, the c-index values for after the first session and within the third session of SWL in the Total-development cohort was 0.712 and 0.723, respectively. In the nomogram utilizing CT information, the respective c-index values were 0.755 and 0.756, which were higher than the c-index values for the nomograms that did not utilize CT information. These results show that our nomograms are highly accurate and discriminate well, similar to previous nomograms [7], and can be convenient models for predicting stone-free probabilities in patients with urinary stones treated with SWL.

Despite the numerous studies and great efforts to discover factors related to SWL outcomes [7, 9, 11, 17–22], a clear general consensus has yet to be reached. Kanao et al. [6] developed a nomogram to predict lithotripsy success based on stone size, location, and the degree of stone burden. Wiesenthal et al. [7] showed stone size, patient age, and SSD as predictors for successful lithotripsy of renal calculi, along with patient BMI, and stone size for successful lithotripsy of ureteral calculi in their nomograms.

In our study, stone location, stone number, stone size (maximal diameter), HU, and grade of HN were significant factors for SFR after SWL of renal and ureteral stones. Sex was an insignificant factor in the multivariate analysis (Odds ratio [OR] = 1.143, 95% CI 0.948–1.378, p-value = 0.163). We, however, included sex in our nomograms based on standard parameters and, as a previous study reported a sex difference [20]. In addition, inclusion of the sex variable modestly improved the predictive accuracy.

CT imaging is considered the gold standard for diagnosing urolithiasis due to its high specificity and sensitivity [7, 17], and in current clinical setting, there is an increasing trend of performing CT scans. In the present study, we therefore analyzed CT-based stone parameters in combination with clinical information.

Consistent with previous studies [9–11, 17, 18, 22], HU was a significant predictor of SFR after SWL in a multivariate analysis in our study. On the other hand, whether HN has an effect on the treatment outcome of SWL is controversial [23, 24]. Seitz et al. [24] reported that the presence or degree of HN in proximal ureteral stones between 4–15 mm does not significantly affect the time to stone clearance or the overall treatment success. Conversely, Ito et al. [4] developed a nomogram using the presence of HN to predict SFR after flexible ureteroscopy (fURS) for renal stones. They suggested that HN causes enlargement of the renal pelvis and calyces, which renders the breaking and basketing of stones more difficult. It is hypothesized that, during SWL, the enlarged renal pelvis and calyces has an effect on targeting, acoustic cavitation, energy density, and other factors [25, 26].

A few recent studies report on the unabated controversial topic of SSD and BMI as predictors of a successful SWL [7, 9, 11, 18, 19, 21, 22]. Despite the positive correlation between BMI and SSD, some reports indicate that BMI and SSD should not be considered as surrogate markers [7]. This has been clarified in some studies as the disparity in body fat distributions between varying sexes and ethnicities [27], and there are new movements using fat mass percentage (FMP) as an alternate variable [19]. In comparison, SSD is gaining support as a significant predictor in various studies [7, 9, 11]. Wiesenthal et al. [7] reported that BMI was not predictive of successful lithotripsy of renal calculi, whereas SSD was prognostic. In this study, SSD failed to be a significant variable. In addition, univariate analysis showed that SSDs for both stone locations (renal vs. ureter) were not significant predictors of SFR for both after the first and within the third session of SWL (S1 Table). We believe that lower mean BMI in our cohort compared to other studies might be contributable to this result.

In current systematic review, Donaldson et al. [2] reported that retrograde intrarenal surgery (RIRS) offers higher SFRs than SWL, particularly for 10–20 mm lower-pole renal stones. However, as RIRS seems to be more invasive than SWL, treatment decisions are not always straightforward. Recently, we developed and validated the modified Seoul National University renal Stone Complexity (S-ReSC) score predicting SFR of renal stones after RIRS [28]. In comparison to the modified S-ReSC, with our nomograms it would be useful to decide the treatment modality for renal stones. Currently, we discourage SWL treatment in patients with lower SFR predicted by our nomograms. Alternatively, we conduct RIRS rather than SWL in considering safety, the cost-benefit ratio, surgeon’s area of expertise, and various patient characteristics in these cases.

This present study also has several limitations that remain to be addressed. First, based on the retrospective nature of the study design, it could be vulnerable to a selection bias. Our data was, however, consecutively collected in three independent institutions with large case numbers, whereby some of these limitations may not have been an issue. Second, the SWL equipment varied across institutions, and there was difficulty in retaining methodological consistency. Nevertheless, any effects on the results were possibly minimal, as recent studies reported that SFRs and reduction in the number of procedures were not machine-dependent [3]. Additionally, a unified shock wave rate and shock wave energy protocol were utilized across our three institutions, and there were no statistically significant differences in them among institutions (data not shown). Third, we developed and validated these nomograms at our branch academic centers with a single practical set-up; however, assessment of the external validity of the nomograms should be conducted using populations of other academic hospitals or community-based centers. Consequently, further investigation through prospective, randomized clinical trials is needed to eliminate several biases, as previously mentioned.

## Conclusion

In summary, we have developed nomograms to predict SFR after the first and within the third session of SWL using the following predictors: sex, stone location, stone number, stone size (maximal diameter), HU, and grade of HN. To the best of our knowledge, these are the first graphical nomograms for the treatment of urinary stones that also have high accuracy and reliability in a large cohort. These nomograms are useful to advise patients on the likelihood of single or multistage SWL treatment.

## Supporting Information

S1 Fig Calibration plots for nomograms predicting stone-free rates after the first and within the third session of shock-wave lithotripsy (SWL) with and without CT information. (A) Total-validation cohort, (B) CT-validation cohort.
(TIF)

S1 Table Univariate logistic regression models for skin-to-stone distance (SSD) according to the stone location (renal vs. ureter) in the CT-development cohorts. (PDF)

## References

[1] Romero V, Akpinar H, Assimos DG. Kidney stones: a global picture of prevalence, incidence, and associated risk factors. Reviews in urology 2010 Spring; 12(2–3):86–96. PMC2931286 20811557

[2] Donaldson JF, Lardas M, Scrimgeour D Stewart F, MacLennan S, Lam TB, et al Systematic Review and Meta-analysis of the Clinical Effectiveness of Shock Wave Lithotripsy, Retrograde Intrarenal Surgery, and Percutaneous Nephrolithotomy for Lower-pole Renal Stones. Eur Urol 2015 4; 67(4):612–6. 10.1016/j.eururo.2014.09.054 25449204

[3] Preminger GM, Tiselius HG, Assimos DG, Alken P, Buck AC, Gallucci M, et al 2007 Guideline for the management of ureteral calculi. Eur Urol 2007 12; 52(6):1610–31. 18074433 10.1016/j.eururo.2007.09.039

[4] Ito H, Sakamaki K, Kawahara T, Terao H, Yasuda K, Kuroda S, et al Development and internal validation of a nomogram for predicting stone-free status after flexible ureteroscopy for renal stones. BJU Int 2015 3; 115(3):446–51. 10.1111/bju.12775 24731157

[5] Smith A, Averch TD, Shahrour K, Opondo D, Daels FP, Labate G, et al A nephrolithometric nomogram to predict treatment success of percutaneous nephrolithotomy. J Urol 2013 7; 190(1):149–56. 10.1016/j.juro.2013.01.047 23353048

[6] Kanao K, Nakashima J, Nakagawa K, Asakura H, Miyajima A, Oya M, et al Preoperative nomograms for predicting stone-free rate after extracorporeal shock wave lithotripsy. J Urol 2006 10; 176(4 Pt 1):1453–6. 16952658 10.1016/j.juro.2006.06.089

[7] Wiesenthal JD, Ghiculete D, Ray AA, Honey RJ and Pace KT A clinical nomogram to predict the successful shock wave lithotripsy of renal and ureteral calculi. J Urol 2011 8; 186(2):556–62. 10.1016/j.juro.2011.03.109 21684557

[8] Demirci D, Sofikerim M, Yalcin E, Ekmekcioglu O, Gulmez I, Karacaqil M. Comparison of conventional and step-wise shockwave lithotripsy in management of urinary calculi. J Endourol 2007 12; 21(12):1407–10. 18044996 10.1089/end.2006.0399

[9] Foda K, Abdeldaeim H, Youssif M, Assem A. Calculating the number of shock waves, expulsion time, and optimum stone parameters based on noncontrast computerized tomography characteristics. Urology 2013 11; 82(5):1026–31. 10.1016/j.urology.2013.06.061 24044913

[10] Kacker R, Zhao L, Macejko A, Thaxton CS, Stern J, Liu JJ, et al Radiographic parameters on noncontrast computerized tomography predictive of shock wave lithotripsy success. J Urol 2008 5; 179(5):1866–71. 10.1016/j.juro.2008.01.038 18353389

[11] Perks AE, Schuler TD, Lee J, Ghiculete D, Chung DG, D’A Honey RJ, et al Stone attenuation and skin-to-stone distance on computed tomography predicts for stone fragmentation by shock wave lithotripsy. Urology 2008 10; 72(4):765–9. 10.1016/j.urology.2008.05.046 18674803

[12] Harrell FE, Califf RM, Pryor DB, Lee KL, Rosati RA. Evaluating the yield of medical tests. JAMA 1982 5; 247(18):2543–6. 7069920

[13] Jeong CW, Jeong SJ, Hong SK, Lee SB, Ku JH, Byun SS, et al Nomograms to predict the pathological stage of clinically localized prostate cancer in Korean men: comparison with western predictive tools using decision curve analysis. Int J Urol 2012 9; 19(9):846–52. 10.1111/j.1442-2042.2012.03040.x 22587373

[14] Partin AW, Mangold LA, Lamm DM, Walsh PC, Epstein JI, Pearson JD.Contemporary update of prostate cancer staging nomograms (Partin Tables) for the new millennium. Urology 2001 12; 58(6):843–8. 11744442 10.1016/s0090-4295(01)01441-8

[15] Kattan MW, Eastham JA, Wheeler TM, Maru N, Scardino PT, Erbersdobler A, et al Counseling men with prostate cancer: a nomogram for predicting the presence of small, moderately differentiated, confined tumors. J Urol 2003 11; 170(5):1792–7. 14532778 10.1097/01.ju.0000091806.70171.41

[16] Jeong CW, Lee S, Jung JW, Lee BK, Jeong SJ, Hong SK, et al Mobile application-based Seoul National University Prostate Cancer Risk Calculator: development, validation, and comparative analysis with two Western risk calculators in Korean men. PLoS One 2014 4; 7;9(4):e94441 10.1371/journal.pone.0094441 24710020 PMC3978062

[17] Hameed DA, Elgammal MA, ElGanainy EO, Hageb A, Mohammed K, El-Taher AM, et al Comparing non contrast computerized tomography criteria versus dual X-ray absorptiometry as predictors of radio-opaque upper urinary tract stone fragmentation after electromagnetic shockwave lithotripsy. Urolithiasis 2013 11; 41(6):511–5. 10.1007/s00240-013-0596-1 23907170

[18] Vella M, Caramia M, Maltese M, Melloni D, Caramia G. ESWL prediction of outcome and failure prevention. Urol Int 2007; 79 Suppl 1:47–50. 17726352 10.1159/000104441

[19] Graversen JA, Korets R, Hruby GW, Valderrama OM, Mues AC, Katsumi HK, et al Evaluation of bioimpedance as novel predictor of extracorporeal shockwave lithotripsy success. J Endourol 2011 9; 25(9):1503–6. 10.1089/end.2010.0687 21815805

[20] Onal B, Tansu N, Demirkesen O, Yalcin V, Huang L, Nquyen HT, et al Nomogram and scoring system for predicting stone-free status after extracorporeal shock wave lithotripsy in children with urolithiasis. BJU Int 2013 2; 111(2):344–52. 10.1111/j.1464-410X.2012.11281.x 22672514

[21] Hatiboglu G, Popeneciu V, Kurosch M, Huber J, Pahernik S, Pfitzenmaier J, et al Prognostic variables for shockwave lithotripsy (SWL) treatment success: no impact of body mass index (BMI) using a third generation lithotripter. BJU Int 2011 10; 108(7):1192–7. 10.1111/j.1464-410X.2010.10007.x 21342413

[22] El-Nahas AR, El-Assmy AM, Mansour O, Sheir KZ. A prospective multivariate analysis of factors predicting stone disintegration by extracorporeal shock wave lithotripsy: the value of high-resolution noncontrast computed tomography. Eur Urol 2007 6; 51(6):1688–93. 17161522 10.1016/j.eururo.2006.11.048

[23] Kirkali Z, Esen AA, Celebi İ, GÜLER C. Are Obstructing Ureteral Stones More Difficult to Treat with Extracorporeal Electromagnetic Shock Wave Lithotripsy?. J Endourol 1993 8; 7(4):277–9. 10.1089/end.1993.7.277 8252017

[24] Seitz C, Fajkovic H, Waldert M, Tanovic E, Remzi M, Kramer G, et al Extracorporeal shock wave lithotripsy in the treatment of proximal ureteral stones: Does the presence and degree of hydronephrosis affect success? Eur Urol 2006 2; 49(2):378–83. 16406241 10.1016/j.eururo.2005.09.022

[25] Yong DZ, Lipkin ME, Simmons WN, Sankin G, Albala DM, Zhonq P, et al Optimization of treatment strategy used during shockwave lithotripsy to maximize stone fragmentation efficiency. J Endourol 2011 9; 25(9):1507–11. 10.1089/end.2010.0732 21834658

[26] Loske AM. The role of energy density and acoustic cavitation in shock wave lithotripsy. Ultrasonics 2010 2; 50(2):300–5. 10.1016/j.ultras.2009.09.012 19819511

[27] Rush E, Plank L, Chandu V, Laulu M, Simmons D, Swinburn B, et al Body size, body composition, and fat distribution: a comparison of young New Zealand men of European, Pacific Island, and Asian Indian ethnicities. N Z Med J 2004 12; 17;117(1207):U1203 15608799

[28] Jung JW, Lee BK, Park YH, Lee S, Jeong SJ, Lee SE, et al Modified Seoul National University Renal Stone Complexity score for retrograde intrarenal surgery. Urolithiasis 2014 8; 42(4):335–40. 10.1007/s00240-014-0650-7 24623504

