# Supplementary figures and images for: Clinical Nomograms to Predict Stone-Free Rates after Shock-Wave Lithotripsy: Development and Internal-Validation
Source: PLoS One. 2016 Feb 18;11(2):e0149333. doi: 10.1371/journal.pone.0149333 (PMC4758663; doi:10.1371/journal.pone.0149333)

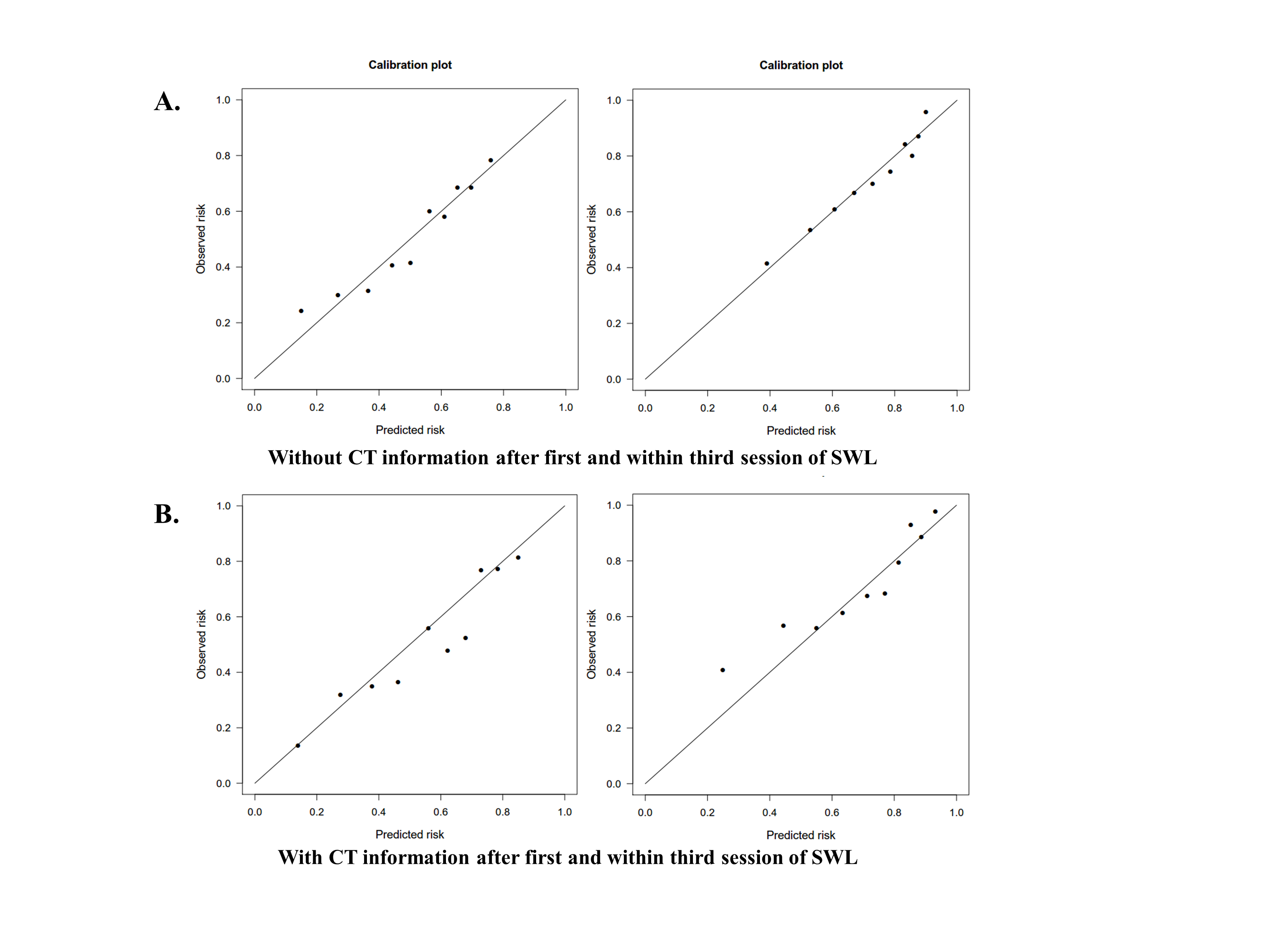

Supplement: S1 Fig — (A) Total-validation cohort, (B) CT-validation cohort. (TIF) [file pone.0149333.s001.TIF]
