# Supplementary material for: Clinical Nomograms to Predict Stone-Free Rates after Shock-Wave Lithotripsy: Development and Internal-Validation
Source: PLoS One. 2016 Feb 18;11(2):e0149333. doi: 10.1371/journal.pone.0149333 (PMC4758663; doi:10.1371/journal.pone.0149333)
Supplement: S1 Table — (PDF) [file pone.0149333.s002.pdf]

S1 Table. Univariate logistic regression models for skin-to-stone distance (SSD) according to the stone location (renal vs. ureter) in the CT-development cohorts.

	OR	95% CI	*p-value*	OR	95% CI	*p-value*
stone-free after first session of Shock-wave lithotripsy						
	Renal stone*			Ureter stone**		
SSD (vertical)	0.985	0.955-1.017	0.365	1.012	0.985-1.040	0.391
SSD (oblique)	0.973	0.940-1.007	0.117	1.001	0.985-1.017	0.893
SSD (horizontal)	0.971	0.935-1.007	0.114	1.006	0.983-1.030	0.602
stone-free within third session of Shock-wave lithotripsy						
	Renal stone*			Ureter stone**		
SSD (vertical)	0.988	0.954-1.024	0.502	1.000	0.971-1.030	0.982
SSD (oblique)	0.988	0.957-1.020	0.459	1.003	0.987-1.019	0.735
SSD (horizontal)	0.990	0.960-1.021	0.526	1.018	0.993-1.044	0.167

* renal stone : upper/mid/lower calyx, pelvis and UPJ stone
** ureter stone : upper/mid/lower ureter stone
